# Understanding the Dynamics of Poliomyelitis Spread in Pakistan

**Published:** 2017-07

**Authors:** Atta Abbas NAQVI, Syed Baqir Shyum NAQVI, Nida YAZDANI, Rizwan AHMAD, Niyaz AHMAD, Fatima ZEHRA

**Affiliations:** 1. Faculty of Pharmacy, Hamdard University, Madinat al Hikmah, Karachi, Pakistan; 2. Dept. of Pharmacy, Clifton Hospital, Karachi, Pakistan; 3. Natural Products and Alternative Medicines, College of Clinical Pharmacy, University of Dammam, Dammam, Saudi Arabia; 4. Dept. of Pharmaceutics, College of Clinical Pharmacy, University of Dammam, Dammam, Saudi Arabia; 5. Dept. of Social Sciences, Shaheed Zulfiqar Ali Bhutto Institute of Science and Technology, Karachi, Pakistan

## Dear Editor-in-Chief

Pakistan’s health care strategy has been geared towards effective polio eradication. By mutual collaboration with WHO, Pakistan has carried out large-scale supplementary immunization activities (SIA) and awareness campaigns to eliminate polio. The results were below par due to some atypical factors and geopolitical issues.

This letter highlights and describes briefly the issues relating to polio spread in Pakistan during last 16 yr. Pakistan is one of countries where polio is still endemic (1). The polio graph has been inconsistent over the years but the cases have escalated incredibly to 306 cases in 2014 (2). The reasons for this public emergency are atypical and in order to understand, one needs to contemplate the circumstances prevailing in the country.

Most of the country is polio-free, however; remote areas of some provinces and administered territories are affected by polio. The polio contagion is concentrated in three regions. This includes the southern region i.e. Karachi division in the province of Sindh, Quetta division which includes Quetta, Killa Abdullah and Pishin districts located in Balochistan Province and Federally Administered Tribal Areas (FATA) region jointly with Peshawar district of neighboring Khyber Pakhtunkhwa Province. The latter two regions border Afghanistan where polio is also endemic. FATA is an administered territory plagued by security issues and lawlessness. Deteriorating security in FATA prompted an internal displacement of local population that spread the polio contagion to other parts of the country. Figures for internally displaced population (IDPs) reported that IDPs settled in the city of Karachi and some rural areas of Punjab and Balochistan Province. Subsequently, all of these areas reported a high number of polio cases the year after (3). This IDP movement had increased likelihood of polio transmission though; due to incomplete figures, this proposition could not be statistically verified (4).

This lawlessness and deteriorated security also hindered routine SIA campaigns resulting in increased prevalence of polio. The health care workers in vaccination teams, which conducted SIA campaigns in FATA, Khyber Pakhtunkhwa, Balochistan and Karachi adjoining areas were routinely attacked by miscreants. Those attacks led to loss of lives that eventually struck down the campaigns (3, 5). These attacks had worst outcome in FATA and Peshawar districts as they became inaccessible for SIA campaigns leading to a large cohort of unimmunized children and hence, a poliovirus reservoir. WHO declared Peshawar as the largest polio reservoir and genetic sequencing results have shown polio strains in found in other areas of the country to be linked to the strain found in FATA and Peshawar (5).

Another reason for polio spread is parental refusal i.e. the refusal of parents to vaccinate their child. It might be due to reasons such as lack of knowledge or attribution of disease, ineffective cold chain management and mistrust towards government. A large segment of population misinterprets the need of polio vaccine as a conspiracy aimed at sterilizing the population to reduce country’s populace and growth rate. At the same time, others believe it contains ingredient forbidden to use in Islam i.e. haram. These misconceptions have been a hurdle in the course of polio eradication. Illiteracy and superstitions regarding polio vaccine have further added to the burden. In addition, Pakistan experience unannounced electricity outages, many vaccines deteriorated due to increased temperature and harsh environment condition that resulted in ineffective vaccination (3, 4). Polio cases were reported in individuals previously immunized which further strengthened the belief of these vaccines as ineffective.

The data of the last 16 yr identify the security issues, attacks on SIA campaigns and subsequent loss of lives, IDPs movements across the country, and parental refusal as four major hurdles in curbing the spread of the disease. The targeting of SIA teams need to be addressed; increasing security arrangements to protect the teams and continuing SIA campaigns must be prioritized. Public perception regarding the polio vaccines and misconception surrounding it must be changed. Public enlightenment is essential for proper understanding of polio disease. It should be focused along with immunization campaigns. Immunizing the IDPs at various checkpoints on the way can help in spreading of polio strain through this channel. Polio has been recently acknowledged as a public emergency in Pakistan. Despite the hurdles, the country is determined to eradicate the disease.
